# Cross-sectional comparison of spiral versus block integrated curriculums in preparing medical students to diagnose and manage concussions

**DOI:** 10.1186/s12909-018-1439-0

**Published:** 2019-01-09

**Authors:** Sarah Fraser, Alexander D. Wright, Paul van Donkelaar, Jonathan D. Smirl

**Affiliations:** 1 Southern Medical Program, Reichwald Health Sciences Centre, University of British Columbia Okanagan, Kelowna, BC Canada; 2 MD/PhD Program, University of British Columbia, Vancouver, BC Canada; 3 Experimental Medicine Program, Faculty of Medicine, University of British Columbia, Vancouver, BC Canada; 4 School of Health and Exercise Sciences, University of British Columbia Okanagan, ART 180, 3333 University Way, Kelowna, V1V 1V7 BC Canada

**Keywords:** Medical education, Curriculum design, Spiral curriculum, Integrated, Concussion

## Abstract

**Background:**

An integrated curriculum is designed to be repetitive yet progressive and the concept has rapidly established itself within medical education. National organizations have recommended a shift to a spiral curriculum design, which uses both vertical and horizontal integration. This study examined differences between the recently implemented integrated spiral (class of 2019) and conventional block (classes of 2016–2018) MD curricula at the University of British Columbia (UBC) with respect to knowledge of concussion.

**Methods:**

Cross-sectional online survey (FluidSurveys: Fluidware, Ottawa, ON), distributed via email to UBC medical students during the 2015–2016 academic year. Questions focused on demographic data, knowledge of concussion definition, and management considerations. Differences in responses across the two groups were assessed using chi-square tests. Ordinal Likert-scale data were analyzed using Mann-Whitney U-Tests. Statistical significance was determined a priori at *p* < 0.05.

**Results:**

One hundred forty eight medical students (57% female) responded with 78 students in the spiral curriculum and 70 students the block curriculum. Important differences between responses from spiral versus block curricula students included: formal exposure to concussion-related educational material (10.8 h spiral vs. 3.95 h block), understanding concussions can occur without direct head impacts (90% spiral vs. 70% block, *X*^2^_1,148_ = 9.41, *p* = 0.002) and identifying long-term consequences (dementia: 90% spiral vs. 66% block, *X*^2^_1,148_ = 12.57, *p* < 0.0001; second impact syndrome: 80% spiral vs. 57% block, *X*^2^_1,148_ = 8.60, *p* = 0.003; Parkinsonism: 47% spiral vs. 17% block, *X*^2^_1,148_ = 14.87, *p* < 0.001). Block students identified the need for a full neurological exam (*X*^2^_1,148_ = 17.63, *p* < 0.001) and had greater clinical exposure to acute concussion (47% block vs. 14% spiral, *X*^2^_1,148_ = 19.27, *p* < 0.001) and post-concussion syndrome (37% block vs. 19% spiral, *X*^2^_1,148_ = 5.91, *p* = 0.015).

**Conclusions:**

The findings from this preliminary study suggest the spiral curriculum design, which emphasizes and revisits clinical competencies, promotes a strong understanding and retention of knowledge in highly prevalent clinical conditions such as concussion.

## Background

The concept of an “integrated curriculum” has become immensely popular in medical education [1]. The belief that effective knowledge acquisition by medical trainees is influenced by the manner in which material is delivered has led integrated curricula – designed to be repetitive yet progressive – to rapidly establish itself within medical schools across North America [1]. Based on many national medical education organization recommendations of strategies to enhance learning [2–5], medical schools all over the world have undergone major curricular evolutions in recent years with increased focus on integrating basic and clinical sciences, facilitating interdepartmental communication, and joint-teaching of courses and content. This focus has led many schools to deviate from the classic curriculum approach – wherein medical trainees complete two preclinical years of lecture-based education in basic science succeeded by two years of clinical training – towards integrated curricula.

In vertically integrated models, students learn both basic and clinical sciences *in parallel* throughout the duration of medical training. One common way of vertically integrating learning is known as Problem-Based Learning (PBL). PBL’s approach is student-centered, focuses on self-directed learning and small group dynamics, and attempts to bring non-clinical material into context enabling students to understand the relevance of underlying scientific knowledge and principles to clinical practice [6, 7]. Extensive research has been conducted comparing traditional learning to PBL to answer those who questioned the effectiveness of this approach. Meta-analyses of twenty years of PBL evaluation students [8–10] report PBL is equal to traditional curriculum in terms of conventional knowledge and there are modest positive outcomes in knowledge retention, motivation, student satisfaction [6, 8, 9]. Horizontal integration, in comparison, combines disciplines (e.g. anatomy, physiology, and biochemistry) into one interdisciplinary course [1, 11]. The most common integrated curriculum using a horizontal strategy targets body systems, or *blocks*, as units of study and often is used in combination with PBL. However, within the *block* system, limited communication between instructors has been shown to create silos of compartmentalized knowledge and gaps in content [12]. As an example, approximately 70% of Canadian medical schools in 2012 did not have concussion-specific curriculum in any study year [13]. As a possible explanation for this lapse in content delivery, Burke et al. suggested concussions are applicable across such a range of disciplines that educators may overlook its coverage, simply believing other disciplines would cover it [13]. As such, the undergraduate medical education program at the University of British Columbia (UBC) has shifted from a *block* to a *spiral* curriculum design, which uses both vertical and horizontal integration to emphasize and revisit clinical themes and competencies in greater detail, reinforcing, integrating and contextualizing important clinical topics (e.g. concussions) [4, 14]. This transition provided an opportunity to assess the extent of exposure to and knowledge of specific topics by medical students training within the two curricula.

Over the course of a career, it is likely every physician will be presented with the diagnosis and/or management of several concussions since, using Canada as an example, approximately one in every thousand experience a concussion each year (~ 38,000/year) [15]. Accordingly, the purpose of this study was to compare medical trainees who trained within a *block* curriculum to trainees within a spiral curriculum with specific respect to their knowledge of diagnosing and managing concussions. In this preliminary study, it was hypothesized that the *spiral* curriculum, suggested to be the most ideal form of integration [1], would promote a better understanding and retention of knowledge in highly prevalent conditions such as concussion.

## Methods

An online survey was distributed to medical students enrolled at the University of British Columbia during the spring semester of the 2015–2016 academic year. First year medical students were enrolled in the new *spiral* curriculum, whereas students in years 2–4 were enrolled in the traditional *block* curriculum. An initial invitation, as well as one reminder was distributed through the Student Affairs office. Study data were collected over three weeks and managed using the Canadian-based online survey platform FluidSurvey (http://fluidsurveys.com). Incoming data were de-identified and the respondents were provided with a study ID number. This study was completely voluntary, and a draw for $50 was used as an incentive to increase participation.

The survey has been used previously to evaluate trainees’ understanding of and exposure to concussion during medical education [16, 17]. Questions were adapted from Boggild and Tator [17] and Donaworth et al. [16], but modified to include an “I don’t know” alternative to more accurately estimate the knowledge of respondents [18]. The first section of the survey (Table 1) gathered information on demographics and personal history of concussion. The next section of the survey assessed medical students’ knowledge of concussion symptoms, diagnosis, and management based on information derived from the most recent International Consensus Statement on Concussion in Sport [19]. The final section of the survey assessed medical students’ educational exposure to concussion.

**Table 1:** Demographics

Group	N	# (%) Female	# (%) with Concussion History
Spiral	78	42 (54)	30 (38)
Block	70	42 (60)	25 (36)
Total	148	84 (57)	55 (37)

*Spiral* students covered concussion midway through their first year (Jan-Feb 2016) and received 10.8 h of concussion related teaching (case-based learning: 5.5 h, small group discussion: 2 h, and lecture: 3.3 h). *Block* students briefly discussed concussion during “Brain and Behaviour”, a nine-week block midway through second year (Jan-Feb 2016 for second year students), and received 3.95 cumulative hours on concussion (small group discussion: 1.75 h and lecture: 2.2 h). Importantly, this number does not quantify the complete exposure of *block* students since they have had a greater propensity for exposure to concussion patients in clinical situations, however the exact time of contact with this patient population varies and therefore was not included in the aforementioned cumulative hours. Notably, learning objectives were nearly identical between curricula with respect to concussion topics related to the survey questions and, in fact, the block curriculum had additional learning objectives related specifically to post-concussion syndrome. Since both *spiral* and *block* curriculum addressed the topic of concussion at the same time of year and the survey was conducted in May 2016, there is unlikely to be a relative advantage of recent knowledge for either group.

Participants were split into two groups – 1st year medical students in the new *spiral* curriculum, and 2nd-4th year medical students in the *block* curriculum – and response frequencies were calculated for each. All statistics were calculated using SPSS Statistics for Macintosh (Version 22.9, IBM Corp., Armonk, NY). Differences in responses across the two groups were assessed using chi-square tests. Ordinal Likert-scale data were analyzed using Mann-Whitney U-Tests. Statistical significance was determined a priori at *p* < 0.05.

## Results

### Demographics

One hundred fifty one surveys out of 1176 were returned (13% response rate), with females comprising 57% of responses. Three data sets were removed from analysis due to apparent lack of honest effort (e.g. selecting the same letter response for all questions), leaving 148 full data sets (Table 1). To examine the potential for non-response bias, responses from late respondents were compared to early respondents based on wave analysis, a commonly used extrapolation approach [20, 21]. No statistically significant differences were found between early and late respondents, suggesting this data was not substantially impacted by nonresponse bias and can be generalized to the sample.

### Definition of Concussion

Overall, 90% of respondents correctly identified the definition of a concussion and 87% correctly labelled concussion as “a brain injury with functional disturbance that cannot be seen on standard imaging”. However, across all medical students only 59% of participants knew that “less than 1/3 of all concussions involve a loss of consciousness”; and just 32% (26% *spiral*; 40% *block*, *X*^2^_1, 148_ = 3.47, *p* = 0.062) were aware that only one symptom is required to make the clinical diagnosis of concussion. Overall, respondents correctly identified many of the most common symptoms of a concussion, with headache, dizziness, and confusion being selected by 99, 94, and 97% of respondents, respectively, with no significant differences evident between the *spiral* and *block* curriculums. However, < 60% of respondents identified vertigo and tinnitus as concussion symptoms (Table 2).

**Table 2:** Knowledge of Concussion

Question	Answer	Spiral Curriculum (% correct)	Block Curriculum (% correct)	*p*-value
What is the definition of concussion? Select the best answer.	A. Loss of consciousness for < 5 min after an impact to the head **B. A complex pathophysiological process affecting the brain, induced by traumatic biomechanical forces** C. A structural brain injury caused by mild traumatic force that transiently decreases cerebral blood flow D. I don’t know	86	94	0.091
Is a concussion a brain injury? Select the best answer.	A. No, as there is no abnormality seen on standard structural neuroimaging B. No, as symptoms are only psychological in nature **C. Yes, as there is a function al disturbance that cannot be seen on standard neuroimaging** D. Yes, as there is structural abnormality seen on standard neuroimaging E. I don’t know	85	88	0.328
Which one of the following is true?	A. A period of unconsciousness is necessary for the diagnosis of a concussion B. Over 2/3 of all concussions involve loss of consciousness (LOC) C. 1/3 to 2/3 of all concussions involve loss of consciousness (LOC) **D. Less than 1/3 of all concussions involve loss of consciousness (LOC)** E. I don’t know	53	67	0.071
Which of the following is a sign or symptom of a concussion? Select all that apply.	A. **Headache**	A. 100	A. 98.6	A. 0.473^a^
	B. Hemiparesis	B. 12.8	B. 8.6	B. 0.406
	C. **Dizziness**	C. 94.9	C. 92.9	C. 0.736^a^
	D. **Confusion**	D. 100	D. 94.3	D. 0.048^a*^
	E. Fixed dilated pupil	E. 21.8	E. 11.4	E. 0.093
	F. **Nausea and**/**or vomiting**	F. 93.6	F. 85.7	F. 0.113
	G. **Vertigo**	G. 55.1	G. 44.3	G. 0.188
	H. **Amnesia**	H. 88.5	H. 94.3	H. 0.211
	I. **Tinnitus**	I. 59	I. 48.6	I. 0.205
	J. **Emotional or personality changes**	J. 79.5	J. 87.1	J. 0.215
	K. Papilledema	K. 11.5	K. 7.1	K. 0.362
	L. Intention tremor	L. 5.1	L. 4.3	L. 1.000^a^
	M. **Fatigue**	M. 80.8	M. 87.1	M. 0.294
	N. **Temporary loss of consciousness**	N. 97.4	N. 94.3	N. 0.422^a^
	O. Prolonged coma	O. 11.5	O. 14.3	O. 0.618
How many symptoms of a concussion are required to diagnose a concussion?	**A. One or more symptoms** B. Three or more symptoms C. Five or more symptoms D. I don’t know	26	40	0.062
Which of the following is true regarding the mechanism of concussion?	A. Direct physical contact to the head is necessary to sustain a concussion B. Localized damage to the brainstem is the cause of a concussion C. Localized damage to the prefrontal cortex is the cause of a concussion D. Localized damage to the hippocampus is the cause of a concussion **E. A whiplash effect to the brain caused by an impact to any part of the body may cause a concussion**	90	70	0.002^*^
What is the appropriate management of concussion? Select all that apply.	A. **Every concussed individual should see a physician**	A. 76.9	A. 80	A. 0.650
	B. A concussed player cannot return to play in the same game or practice if examined by a physician	B. 60.3	B. 41.4	B. 0.835
	C. **A stepwise increase in exercise and activity if symptomatic**	C. 67.9	C. 77.1	C. 0.212
	D. **Physical rest is always recommended after a concussion**	D. 93.6	D. 84.3	D. 0.069
	E. **Mental rest is always recommended after a concussion**	E. 92.3	E. 87.1	E. 0.299
	F. **Signs and symptoms should be monitored for increasing severity**	F. 92.3	F. 91.4	F. 0.845
	G. **Full neurological exam at initial assessment is recommended**	G. 50	G. 82.9	G. < 0.001^*^
	H. The standard mini mental status exam at initial assessment is an adequate cognitive test for concussion	H. 53.8	H. 14.3	H. < 0.001^*^
	I. MRI of the brain is mandatory	I. 0	I. 1.4	I. 0.473^a^
	J. CT of the brain is mandatory	J. 7.7	J. 4.3	J. 0.500^a^
What are some “red flags” that may predict the potential for more prolonged symptoms and may influence your investigation and management of concussion? Select all that apply	A. Nose bleed	A. 16.7	A. 25.7	A. 0.177
	**B. Prolonged loss of consciousness**	B. 98.7	B. 94.3	B. 0.189^a^
	**C. Number and duration of symptoms**	C. 88.5	C. 92.9	C. 0.362
	**D. Age**	D. 93.6	D. 81.4	D. 0.024^*^
	**E. Repeated concussions occurring with progressively less impact force**	E. 79.5	E. 85.7	E. 0.229
	**F. Slower recovery after each successive concussion**	F. 85.9	F. 91.4	F. 0.292
	**G. Repeated concussions over time**	G. 92.3	G. 90	G. 0.620
	**H. Concussions close together in time**	H. 88.5	H. 90	H. 0.763
	I. Being hit on the left side of the head	I. 2.6	I. 7.1	I. 0.256^a^
What are the long-term consequences of repetitive concussive injury? Select all that apply.	**A. Dementia**	A. 89.7	A. 65.7	A. < 0.001^*^
	**B. Depression**	B. 87.2	B. 91.4	B. 0.406
	**C. Headaches**	C. 85.9	C. 87.1	C. 825
	D. Increased risk of hemorrhagic stroke	D. 29.5	D. 28.6	D. 0.902
	**E. Death or disability with second concussion before recovery from a first concussion**	E. 79.5	E. 57.1	E. 0.003^*^
	F. Increased risk of schizophrenia	F. 20.5	F. 17.1	F. 0.601
	**G. Prolonged fatigue**	G. 59	G. 65.7	G. 0.399
	**H. Impairment of concentration and memory**	H. 85.9	H. 87.1	H. 0.825
	**I. Parkinsonism**	I. 47.4	I. 17.1	I. < 0.001^*^
	**J. Chronic traumatic encephalopathy**	J. 73.1	J. 50	J. 0.004^*^

*P* values determined with chi-square test. ^*^Indicates significant results. Correct answers in boldface.

^a^In samples where the expected frequencies were < 5, Fisher’s Exact Test was used

Interestingly, when identifying the mechanism of concussion, 90% of those in the *spiral* curriculum, compared to 70% of those in the *block* curriculum, responded that a concussion could be caused by a whiplash effect to the brain caused by an impact to any part of the body (*X*^2^_1, 148_ = 9.41, *p* = 0.002).

### Concussion management and sequelae

Medical students at UBC seem to be generally aware of appropriate concussion management strategies. 78% of respondents responded that every concussed individual should see a physician and 90% understand that both physical and mental rest is recommended acutely post-concussion. It was also well understood that neuroimaging adds little to the assessment of concussion as demonstrated by the 1 and 6% of respondents who indicated “MRI of the brain is mandatory” and “CT of the brain is mandatory”, respectively. Potential red flags such as prolonged loss of consciousness, repeated concussions over time, and number and duration of symptoms were also correctly identified 97, 91, and 91% of the time respectively. Of note, 50% from the *spiral* curriculum and 83% from the *block* curriculum correctly identified the recommendation for a full neurological exam (*X*^2^_1, 148_ = 17.63, *p* < 0.001); 54% of spiral students believed that a mini-mental status was adequate compared to 14% of block students (*X*^2^_1, 148_ = 25.33, *p* < 0.001).

When asked to identify long-term consequences of repetitive concussive injuries, students in the *spiral* curriculum identified dementia (*X*^2^_1, 148_ = 12.57, *p* < 0.001), second impact (*X*^2^_1, 148_ = 8.60, *p* = 0.003), Parkinsonism (*X*^2^_1, 148_ = 14.87, *p* < 0.001), and CTE (*X*^2^_1, 148_ = 8.35, *p* = 0.004) significantly more frequently than those in the *block* curriculum (refer to Table 2).

### Exposure

*Spiral* students were exposed to concussion education through lectures, case-based learning, and group discussion seminars adding up to 10.8 h of related or specific concussion education. This greatly exceeded *block* curriculum exposure, which provided 3.95 h (3h seminar and didactic lecture). Both year 1 (*spiral*) and 2 (*block*) students were exposed to the theory of concussion at the same time (Jan-Feb, 2016) while *block* students from year 3 and 4 would have been further distanced from their formal education in this topic. Formal education however, does not encompass all of the learning opportunities for medical trainees. This is demonstrated by *block* curriculum students who had much greater exposure to patients with acute phase concussion (*block*: 47%, *spiral*: 14%, *X*^2^_1, 148_ = 19.27, *p* < 0.001) and post-concussion syndrome (*block*: 37%, spiral: 19%, *X*^2^_1, 148_ = 5.91, *p* = 0.015), and is consistent with differences in cumulative clinical exposure between groups. The additional clinical exposure for the advanced *block* trainees likely offsets a portion of the additional seminar hours for the *spiral* trainees resulting in a less disparate total exposure between groups.

### Desire versus knowledge

When asked to self-rank their knowledge of concussion on a scale of 1 (inadequate) – 100 (completely adequate), *Spiral* students scored 41 ± 22.9 while *Block* students scored 43 ± 19.4 (Table 3). This was not statistically significant as determined by a Mann-Whitney U test (*U* = 2863, *z* = 0.511, *p* = 0.609). Similarly, when asked to rank their desire to learn more about concussions on a scale of 1–100, from Not at All to Very Much, students ranked 75.3 ± 24 and 75.3 ± 18.4 for the *Spiral* and *Block* curriculums, respectively (*U* = 2778.5, *z* = 0.187, *p* = 0.852) (Table 3). Both groups identified textbooks and the website Up-to-Date as the resources most likely employed for finding more information.

**Table 3:** Self-ranking of concussion knowledge and desire to learn

Question	Spiral Curriculum Mean ± SD	Block Curriculum Mean ± SD	*p*-value
How would you self-rank your knowledge about concussions? (average)	41 ± 22.9	43 ± 19.4	0.609
Are concussions something you want to learn more about as part of your medical curriculum? (average)	75.3 ± 24.4	75.3 ± 18.4	0.852

P values determined with Mann-Whitney U test

## Discussion

This preliminary study aimed to compare students training within a *block* curriculum to students within a *spiral* curriculum with respect to their knowledge of diagnosing and managing concussion. The vast majority of students were able to correctly identify the definition of concussion, the most common concussion symptoms, and appropriate management steps indicating both curricular structures have merit. The study results however, demonstrated several relative advantages of the *spiral* curriculum, including augmented formal exposure to concussions and filling knowledge gaps related to injury mechanisms and potential long-term consequences (Table 2) despite substantially less clinical exposure.

Though the results of this preliminary study suggest a number of positive signs regarding concussion education within a single Canadian medical school (UBC), areas for improvement were also identified. In studies on which the current survey was based, Boggild and Tator (2012) and Donaworth (2016) found specific misconceptions around long-term consequences of concussions with half of the medical trainees not recognizing CTE or second impact syndrome as possibilities. While students from the *block* curriculum followed this trend with only 50% recognizing CTE and 43% recognizing Second Impact Syndrome as long-term outcomes, 73 and 80% of *spiral* curriculum students recognized CTE and Second Impact Syndrome, respectively. Prolonged fatigue was poorly recognized by both groups however (*block:* 55%; *spiral*: 66%, *X*^2^_1, 148_ = 0.71, *p* = 0.399), at rates only slightly higher than previous studies (43.8%) [11]. Similarly, Parkinsonism was poorly recognized in our sample (*block*: 66%; *spiral*: 59%, *X*^2^_1, 148_ = 14.87, *p* < 0.001), but at a rate appreciably higher than reported in Donaworth’s results (29.3%) [11]. It is possible this may be influenced by recent media attention on concussions, including Muhammad Ali’s journey with Parkinson’s Disease that preceded his recent passing. Of note, 22% of all students did not report, “every concussed individual should see a physician”. Similar to the 24% of incorrect responses from Boggild and Tator’s (2012) study, this may reflect a troubling idea that medical professionals do not believe concussions represent sufficiently serious injury to warrant medical attention.

It is important to note this survey identified a distinct lack of understanding of the diagnostic criteria for a concussion. Over half of the students from both *block* and *spiral* curricula were unaware concussions may be diagnosed with only one symptom and that “less than 1/3 of all concussion involve loss of consciousness”. Concussion remains a clinical diagnosis, and physicians need to have the knowledge and awareness of concussion to enable appropriate diagnosis and subsequent management of such patients, including mitigation of post-concussive sequelae [22]. Positive findings on any subjective symptoms, physical signs, behavioural changes, cognitive impairment, or sleep disturbances should prompt concussion to be considered in a differential diagnosis, and appropriate management steps should subsequently be taken [19]. Medical students in the current study understood neuroimaging typically does not add to the assessment of concussion, unless intracerebral or structural lesions are suspected on clinical grounds [19]. These scenarios would be associated with further “red flag” symptoms such as prolonged loss of consciousness or focal neurological deficit, of which the majority of students were well aware [19].

Although students in the *spiral* curriculum only had one year of medical training at the time of this study, they performed better than those in the *block* curriculum in identifying the mechanism of injury and potential long-term sequelae of concussion. This is in the context of more formal but less clinical exposure time though students in both academic learning environments likely had similar overall exposure time to gain knowledge about concussions. These findings support the evolution of *spiral* curricula in which clinically relevant information is integrated at an earlier point in training. While *block* curriculum students were considerably more likely to have seen a patient with acute concussion (*block:* 47%, *spiral:* 14%, *X*^2^_1, 148_ = 19.28, *p* < 0.001) *or* post-concussive symptoms (*block*: 37%, *spiral*: 19%, *X*^2^_1, 148_ = 5.91, *p* = 0.015), this can be attributed to the additional clinical training in years 3 and 4 of the MD program. This difference may also explain why those in the *block* curriculum, who had more frequently participated in the care of concussion patients, had a better understanding that concussion evaluation requires a full neurological exam at initial assessment (*block*: 82.9%, *spiral*: 50%, *X*^2^_1, 148_ = 5.91, *p* < 0.001) and that a mini-mental status exam is insufficient for such purposes (*block*: 14%, *spiral*: 54%, *p* < 0.001). We are also optimistic of even further progression of spiral student knowledge based on the very nature of the *spiral* curriculum’s repeated exposure to the same subject area at different time points in the curriculum. An example includes the theory of dementia, addressed at the end of year two in the *spiral* curriculum, of which prior concussion may be a risk factor.

Brauer et al. have recommended the spiral approach as an ideal curriculum model for enhancing the knowledge base of medical trainees [1, 4]. Within UBC’s evolutionary approach, basic clinical presentations follow a natural progression towards more complex patient profiles across the educational pipeline [14]. Just as previous studies determined modest superiority of the integrated PBL model over traditional curricula [8], our study has found that further increasing basic and clinical science integration, in this case through a *spiral* curriculum, allowed students to perform just as well and sometimes better than students in the *block* curriculum. Although this study focuses on the topic of concussion education, it is likely the same principles would be of value if applied to other prominent conditions or topics within medical education.

Many of the concerns that prompted the development of the PBL curriculum in medical schools are still echoed today and related student-centered interventions certainly have their skeptics [23, 24]. Similar to the changes toward PBL, the spiral curriculum style has several disadvantages and it is important to also acknowledge that creating a spiral curriculum can be time consuming and resource-intensive with logistical challenges that include synchronous presentation of material, detailed mapping of content, and preservation of the basic sciences [1]. While not the focus of this paper, there are many publications available reviewing integrated curricular projects discussing challenges and opportunities [1, 14, 25, 26].

This study has clear limitations; data were collected from a single institution and may not be generalizable to all medical schools. With a 13% response rate, a potential response bias may exist, whereby students who felt they knew more about concussion or those who were displeased with the amount of education they were receiving may have been more inclined to complete the survey. However, no statistically significant differences were found between early and late respondents, suggesting the conclusions drawn from the data have not been substantially impacted by nonresponse bias [19]. In addition, this was not a strict exam setting and it is possible that student’s may have looked up answers. We believe this is unlikely since there was no incentive for correct answers and no marks given at the end of the survey. Finally, the timing of this study within each curriculum is an important methodological issue. If the *block* students were exposed to concussion long before the *spiral* students these results may have been explained by the advantage of having more recent exposure to the topic. However, both *spiral* and 2nd year *block* curriculum addressed the topic of concussion at the same time of year and 3rd and 4th year block students had considerably more clinical exposure to concussion, an advantage over the spiral students, making it unlikely that any advantage of recent knowledge exists for the *spiral* cohort. We recognize that the *spiral* curriculum received additional formal exposure to the material and this may have impacted the results, but again the *block* curriculum students had significantly more clinical exposure to balance this out. It could also be argued that we do not have a true picture of the long-term effect of either the *block* or the *spiral* curriculum and that differences may be more or less apparent at a later stage in their careers. In the future, this study will be expanded with both cohorts in their final year to confirm the current findings. It would also be of value to study comparative questions in exam banks completed under formal exam settings to look for difference between cohorts. Future studies are warranted to evaluate student perceptions of their training and readiness for practice as well as if the *spiral* curriculum continues to be effective in remedying the reported gaps in medical trainee knowledge with respect to concussions and other clinical disorders, with the overall goal of determining whether changes in curricula translate into better patient care.

## Conclusions

Many medical schools are redesigning their programs creating a meaningful opportunity for the integration of education and more research into the pros and cons of a spiral curriculum. The findings from this study suggest that the *spiral* curriculum design, which emphasizes, integrates, and contextualizes clinical competencies, performs at least as well as the *block* curriculum of distinct courses on body systems with respect to one clinical topic; however, how this learning model applies to other topics has yet to be elucidated. At least in a preliminary manner, the spiral curriculum has the potential to promote a strong understanding and retention of knowledge in highly prevalent conditions such as concussion.
